# Supplementary material for: Recurrence of Idiopathic Membranous Nephropathy in the Kidney Allograft: A Systematic Review
Source: Biomedicines. 2024 Mar 26;12(4):739. doi: 10.3390/biomedicines12040739 (PMC11048506; doi:10.3390/biomedicines12040739)
Supplement: Supplementary file 1 [file biomedicines-12-00739-s001.zip › Supplementary Materials S2.pdf]

## Supplementary Material 2

**Table S1.** Search strategy in PubMed database.

| | **PUBMED (n=274)** | |
|---|---|---|
| **Search** | **Query** | **Results** |
| **#7** | Search: (((kidney) AND (membranous AND nephropathy)) AND (posttransplantation)) AND (recurrence) Sort by: Most Recent | **41** |
| **#6** | Search: (#5) AND (rituximab) Sort by: Most Recent | **133** |
| **#5** | Search: (((kidney OR renal) AND (transplantation OR transplant OR allograft)) AND (recurrence OR recurrent)) AND (membranous nephropathy OR nephritis) Sort by: Most Recent | **1532** |
| **#4** | Search: ((allograft) AND (membranous nephritis)) AND (recurrence) Sort by: Most Recent | **173** |
| **#3** | Search: (((kidney transplantation) AND (membranous nephropathy)) AND (recurrence)) AND (rituximab) Sort by: Most Recent | **29** |
| **#2** | Search: ((kidney transplantation) AND (membranous nephropathy)) AND (post transplantation) Sort by: Most Recent | **83** |
| **#1** | Search: ((kidney transplantation) AND (membranous nephropathy)) AND (recurrence) Sort by: Most Recent | **209** |

**Table S2.** Search strategy in SCOPUS database.

| SCOPUS (n=272) | | |
|---|---|---|
| **ID** | **Query** | **Documents** |
| #7 | ( ALL ( kidney ) AND ALL ( membranous AND nephropathy ) AND ALL ( posttransplantation ) A ND ALL ( recurrence ) ) | **180** |
| #6 | ( ( ALL ( kidney AND transplantation ) AND ALL ( membranous AND nephropathy ) AND ALL ( recurrence ) AND ALL ( rituximab ) ) ) AND ( ( ALL ( kidney OR renal ) AND ALL ( tranplantati on OR transplant OR allograft ) AND ALL ( recurrence OR recurrent ) AND ALL ( membranous AND nephropathy OR nephritis ) ) ) | **406** |
| #5 | ( ALL ( kidney AND transplantation ) AND ALL ( membranous AND nephropathy ) AND ALL ( r ecurrence ) AND ALL ( rituximab ) ) | **507** |
| #4 | ( ALL ( kidney OR renal ) AND ALL ( tranplantation OR transplant OR allograft ) AND ALL ( re currence OR recurrent ) AND ALL ( membranous AND nephropathy OR nephritis ) ) | **1852** |
| #3 | ( ALL ( allograft ) AND ALL ( membranous AND nephritis ) AND ALL ( recurrence ) ) | **439** |
| #2 | ( ALL ( kidney AND transplantation ) AND ALL ( membranous AND nephropathy ) AND ALL ( posttransplantation ) ) | **301** |
| #1 | ALL ( "kidney AND transplantation" AND "membranous AND nephropathy" AND "recurrence" ) | **1400** |

**Table S3.** Search strategy in Web of Science database.

| WEB OF SCIENCE (n=149) | | |
|---|---|---|
| **Set** | **Results** | **Saved History** |
| #6 | **60** | (#5) AND ALL=(rituximab) |
| #5 | **494** | (((ALL=(kidney OR renal)) AND ALL=(transplantation OR transplant OR allograft)) AND ALL=(recurrence OR recurrent)) AND ALL=(membranous nephropathy OR nephritis) |
| #4 | **23** | ((ALL=(allograft)) AND ALL=(membranous nephritis)) AND ALL=(recurrence) |
| #3 | **26** | (((ALL=(kidney transplantation)) AND ALL=(membranous nephropathy)) AND ALL=(recurrence)) AND ALL=(rituximab) |
| #2 | **11** | (((ALL=(kidney)) AND ALL=(membranous AND nephropathy)) AND ALL=(posttransplantation)) AND ALL=(recurrence) |
| #1 | **103** | ((ALL=(kidney transplantation)) AND ALL=(membranous nephropathy)) AND ALL=(recurrence) |

**Table S4**. Demographic characteristics of transplanted patients with iMN recurrence.

| | |
|---|---|
| Age (mean, years) n=139 | 47.82 |
| Men (n) | 93/130 (71.54%) |
| Women (n) | 37/130 (28.46%) |
| Living donors (n) | 89/123 (72.36%) |
| Deceased donors (n) | 34/123 (27.64%) |
| ABO Incompatibility (n) | 2/33 (6.07%) |
| Biopsy with iMN in native kidney (n) | 128/130 (98.46%) |
| Induction therapy: | |
| • ATG (n) | 41/58 (70.68%) |
| • Basiliximab (n) | 13/34 (38.23%) |
| • Alemtuzumab (n) | 4/18 (22.22%) |
| • OKT3 (n) | 0/5 (0%) |
| • Plasmapheresis* (n) | 1/15 (6.67%) |
| • Rituximab* (n) | 2/33 (6.07%) |
| Maintenance therapy: | |
| • Steroids (n) | 61/71 (85.92%) |
| • MPA (n) | 50/62 (80.64%) |
| • Tacrolimus (n) | 83/113 (73.45%) |
| • Cyclosporin (n) | 30/113 (26.55%) |
| • Azathioprine (n) | 7/20 (35%) |
| Diagnostic biopsy for recurrent iMN: | |
| • Per- protocol (n) | 45/134 (33.59%) |
| • Clinical indications (n) | 89/134 (66.41%) |
| Serum creatinine at recurrence (mean, mg/dl) n=97 | 2.00 |
| Proteinuria at recurrence (mean, g/24h) n=117 | 3.97 |
| Treatment of recurrent iMN with Rituximab: ¥ (n) | 55/115 (47.82%) |
| • 4 doses of 375 mg/m$^2$ per week (n) | 8 |
| • Single dose of 1gr (n) | 2 |
| • 2 doses of 1 gr with an interval of 2 weeks (n) | 19 |
| • 1-2 doses of 375 mg/m$^2$ with an interval of 2 weeks (n) | 6 |

*: ABO Incompatibility
¥: not mentioned in 20 patients

**Table S5.** Quality assessment using the ROBINS-E tool.

| Study | Confounding | Classification of exposures | Selection of participants | Deviations from intended exposures | Missing Data | Measurement of outcomes | Selection of the reported result | Overall bias |
|---|---|---|---|---|---|---|---|---|
| **Couchoud et al. 1995** | Some Concerns | Low | Low | Low | Low | Low | Low | **Moderate** |
| **Briganti et al. 2002** | Low | Low | Low | Low | Low | Low | Low | **Moderate** |
| **Ibrahim et al. 2006** | Low | Low | Low | Low | Low | Low | Low | **Low** |
| **Moroni et al. 2010** | Low | Low | Low | Some Concerns | Low | Low | Low | **Moderate** |
| **Sprangers et al. 2010** | Low | Low | Low | Some Concerns | Low | Low | Low | **Moderate** |
| **Debiec et al. 2011** | Some Concerns | Low | Low | Low | Low | Low | Low | **Moderate** |
| **Rodriguez et al. 2012** | Some Concerns | Some Concerns | Low | Some Concerns | Low | Low | Low | **Moderate** |
| **Kennedy et al. 2013** | Some Concerns | Low | Low | Some Concerns | Low | Low | Low | **Moderate** |
| **Kattah et al. 2015** | Low | Low | Low | Some Concerns | Low | Low | Low | **Moderate** |
| **Quintana et al. 2015** | Some Concerns | Low | Low | Some Concerns | Some Concerns | Low | Low | **Moderate** |
| **Grupper et al. 2016** | Some Concerns | Low | Low | Some Concerns | Low | Low | Low | **Moderate** |
| **Gupta et al. 2016** | Low | Low | Low | Some Concerns | Low | Low | Low | **Moderate** |

**Table S6.** Graft survival in transplanted patients with recurrent iMN.

| Study | Transplanted patients due to iMN (n) | Recurrent iMN in renal allograft (n)+ | Graft loss due to recurrent iMN (n) | Control transplanted patients with no iMN recurrence (n) | Graft loss due to any other cause (n) |
|---|---|---|---|---|---|
| **Couchoud et al. 1995** | 18 | 5 | 1 (20%) | 14 | 5 (35.71%) |
| **Briganti et al. 2002** | 81 | 5 | -* | 76 | -¥ |
| **Ibrahim et al. 2006** | 19 | 4 | 0 (0%) | Group 1: transplant patients due to various types of GN with rapid discontinuation of corticosteroids (n=105)<br>Group 2: transplanted patients for non-GN diseases with rapid discontinuation of corticosteroids (n=439)<br>Group 3: historical control group of transplanted patients due to various types of GN with long-term steroid-based immunosuppressive therapy (n=260) | -¥ |
| **Moroni et al. 2010** | 35 | 12 | 6 (50%) | 23 | 11 (47.82%) |
| **Sprangers et al. 2010** | 34 | 15 | 2 (13.33%) | 19 | 4 (21.05%) |
| **Debiec et al. 2011** | 10 | 11+ | 6 (54.54%) | 6 | -¥ |
| **Rodriguez et al. 2012** | 18 | 18 | 0 (0%) | 8 | 0 (0%) |
| **Kennedy et al. 2013** | 32 | 13+ | 7 (53.84%) | 23 | 6 (26.08%) |
| **Kattah et al. 2015** | 26 | 18 | 2 (11.11%) | 8 | 2 (25%) |
| **Quintana et al. 2015** | 21 | 7 | 1 (14.28%) | 14 | -¥ |
| **Grupper et al. 2016** | 63 | 30 | 5 (16.66%) | 33 | 6 (18.18%) |
| **Gupta et al. 2016** | 16 | 6 | 0 (0%) | 10 | 1 (10%) |
| **Total** | **373** | **144** | **30 (21.59%)**** | **234** | **35 (25.36%)***** |

+: refers to number of grafts as some patients received >1 kidney transplants.
*: only the hazard ratio is presented in this study
¥: number of grafts lost in control patients not reported.
**: for n=139 grafts
***: for n=138 control patients
GN: glomerulonephritis

**Table S7.** Graft survival between patients with iMN recurrence that were treated with rituximab and those that received no specific treatment.

| Study | Number of patients | | | | | |
|---|---|---|---|---|---|---|
| | Recurrence iMN + RTX | | Recurrence iMN + no RTX | | No Recurrence | |
| | Graft loss | Survival | Graft loss | Survival | Graft loss | Survival |
| **Couchoud et al. 1995*** | -* | -* | 1 (25%) | 4 | 5 | 9 |
| **Moroni et al. 2010** | 1 (50%) | 1 | 5 (50%) | 5 | 11 | 12 |
| **Sprangers et al. 2010** | 0 (0%) | 4 | 2 (18.19%) | 9 | 4 | 15 |
| **Rodriguez et al. 2012** | 0 (0%) | 8 | 0 (0%) | 10 | 0 | 8 |
| **Kennedy et al. 2013** | 0 (0%) | 1 | 7 (58.34%) | 5 | 6 | 17 |
| **Kattah et al. 2015** | 3 (27.28%) | 8 | 0 (0%) | 5 | 2 | 6 |
| **Quintana et al. 2015** | 1 (25%) | 4 | 0 (0%) | 3 | -¥ | -¥ |
| **Grupper et al. 2016** | 3 (17.64%) | 14 | 2 (15.39%) | 11 | 6 | 27 |
| **Gupta et al. 2016** | 1 (16.67%) | 5 | 0 (0%) | 0 | 1 | 9 |

RTX: Rituximab, iMN: idiopathic membranous nephropathy
* No rituximab was given
¥ number of grafts lost in control patients not reported
